# Editorial: Multi-Disciplinary Approaches to Plant Responses to Climate Change

**DOI:** 10.3389/fpls.2022.876432

**Published:** 2022-03-16

**Authors:** Varodom Charoensawan, Sandra Cortijo, Mirela Domijan, Sónia Negrão

**Affiliations:** ^1^Department of Biochemistry, Faculty of Science, Mahidol University, Bangkok, Thailand; ^2^Integrative Computational Bioscience Center, Mahidol University, Bangkok, Thailand; ^3^Systems Biology of Diseases Research Unit, Faculty of Science, Mahidol University, Bangkok, Thailand; ^4^UMR5004 Biochimie et Physiologie Moléculaire des Plantes, Montpellier, France; ^5^Department of Mathematical Sciences, University of Liverpool, Liverpool, United Kingdom; ^6^School of Biology and Environmental Science, College of Science, University College Dublin, Dublin, Ireland

**Keywords:** climate change, systems biology, omics analyses, interdisciplinary research, bioinformatics

In August 2021, the Intergovernmental Panel on Climate Change (IPCC, 2021) released a new report on the anthropogenic effects of climate change. Climate change is causing a steady rise of global surface temperature and atmospheric CO_2_ concentration, increasing the frequency and intensity of temperature extremes, causing heat waves, heavy precipitation, and, in some regions, agricultural and ecological droughts (Schiermeier, 2018). Such knock-on effects of climate change have a dire impact on plants, affecting plant development and reproduction, impacting natural vegetation and agriculture. Hence, to overcome changes in their environment, plants must adapt swiftly and respond to changes in their natural settings or else face extinction.

Environmental cues, such as progressive changes in photoperiod, or in day and nighttime temperatures, allow the plant to make intricate developmental decisions to negotiate diverse environmental conditions through different seasons (Wilczek et al., 2009; Zhao et al., 2020). On the other hand, severe and sudden environmental changes lead to stresses that the plants must quickly respond and adapt to, in order to survive (Balasubramanian et al., 2006; Berg et al., 2019). Thus, it is crucial for us to continue to advance our overall understanding of how plants respond and adapt to environmental changes, and to work toward improving their resilience in face of the unpredictability of climate change.

Several decades of studies, especially in model plants such as *Arabidopsis*, have demonstrated the complexity of molecular mechanisms, that plants employ to perceive and respond to the ever-changing environments, highlighting the need for large-scale data analysis, integration, and comparison of multiple related data sets, as well as for the combination of multidisciplinary approaches to address such complex problems, e.g., Jung et al. (2016), Cortijo et al. (2017), Awlia et al. (2021), and Sriden and Charoensawan (2022). More recently, latest technological developments and improvements have allowed the acquisition of phenomics, physiological, transcriptomic, epigenomic, and microscopic data, as well as comprehensive data integration, in non-model crops and agricultural plants, as summarized in Zenda et al. in this Research Topic, and also other recent reviews (Wee and Dinneny, 2010; Scossa et al., 2021; Yang et al., 2021).

This Research Topic highlights multidisciplinary efforts that tackle responses to a wide variety of environmental changes associated with climate change, including cold (Wang et al.), drought (Wang et al.; Liu et al.) and even mineral deficiency (Zhang et al.). Interestingly, all the articles presented in this issue focus on non-model crop plants, highlighting the importance of applying omics and multi-omics techniques beyond conventional model plant species, as well as showing that omics techniques have been fully developed to be successfully translated in a wide variety of crop species.

In this Research Topic, Wang et al. combined transcriptomics and metabolomics to explore genes and metabolites that are involved in responses to cold and drought stresses in *Poa crymophila Keng*, a variation of gramineous forage grass widely distributed in the Qinghai-Tibet Plateau. The authors were able to identify the phenylpropanoid pathway, among a few others, as the key mechanism that allows the plant to adapt to these harsh environments. Using transcriptomics in combination with physiological analyses, Liu et al. have characterized morphological and physiological responses against chilling stress in 14 pumpkin (*Cucurbita moschata*) varieties, and identified differentially expressed genes in the leaves and the roots, notably, several in the α-linolenic acid biosynthesis pathway. Zhang et al. used a combination of phenomics, statistical methods and physiological measurements, including photosynthetic indicators to monitor the root architecture phenotypes of cotton (*Gossypium hirsutum*) under Phosphorus deficiency. The authors presented RhizoPot, an improvised *in situ* root phenotyping observation device, and found that several changes in root surface area are important indicators of cotton root phenotypes under low phosphorus. Finally, Zenda et al. reviewed the recent developments in omics that can help us toward crop improvement and breeding more resilient plants.

In summary, the articles presented in this Research Topic further reiterate that multidisciplinary approaches have already shown their importance, and are on their way to becoming indispensable in our quest to understand how the model plants as well as non-model crops respond to a wide range of stress conditions. This will in turn pave the way for the rational design and development of crops that are resilient to various stresses associated with climate change.

## Author Contributions

VC, SC, MD, and SN wrote and revised the article. All authors contributed to the article and approved the submitted version.

## Funding

Work in the laboratory of VC was supported by the mid-career researcher grant from National Research Council of Thailand (NRCT) and Mahidol University (NRCT5-RSA63015-24), and Mahidol University's Postdoctoral Fellowship (MU-PD_2021_09). SN acknowledges the funding from Science Foundation Ireland through the SFI President of Ireland Future Research Leaders under Grant No. 18/FRL/6197.

## Conflict of Interest

The authors declare that the research was conducted in the absence of any commercial or financial relationships that could be construed as a potential conflict of interest.

## Publisher's Note

All claims expressed in this article are solely those of the authors and do not necessarily represent those of their affiliated organizations, or those of the publisher, the editors and the reviewers. Any product that may be evaluated in this article, or claim that may be made by its manufacturer, is not guaranteed or endorsed by the publisher.
